# Meta-analysis of the clinicopathologic features of endometrial cancer molecular staging

**DOI:** 10.3389/fonc.2024.1510102

**Published:** 2025-01-07

**Authors:** Xiaoxia Yin, Bing Luo, Yong Li

**Affiliations:** ^1^ The First Affiliated Hospital of Hebei North University, Zhangjiakou, Hebei, China; ^2^ Department of Pathology, The First Affiliated Hospital of Hebei North University, Zhangjiakou, Hebei, China; ^3^ Department of Imaging, The First Affiliated Hospital of Hebei North University, Zhangjiakou, Hebei, China

**Keywords:** endometrial cancer, molecular typing, TCGA, pathologic features, meta-analysis

## Abstract

**Objective:**

The 2013 TCGA identified four molecular subgroups of endometrial cancer; however, the data results for most of the pathological features were varied and of low value for clinical application. Therefore, a meta-analysis of articles related to the clinicopathological features of molecular typing was performed to observe how the prevalence of the four subgroups varied across different pathological features and whether they were associated with certain specific pathological features and to understand how molecular typing may influence current pathological assessments.

**Methods:**

PubMed, Embase, Web of Science, CNKI, Wanfang, and VIP were searched from the time of library construction until May 2024, and the following data were extracted: histological type, FIGO grade, FIGO stage, LVSI, depth of muscularis propria infiltration, and lymph node status of each TCGA group. Two reviewers used the Cochrane Diagnostic Research Scale assessment, and the data were analyzed using Review Manager 5.4.1 and Stata 14.0.

**Results:**

Fourteen diagnostic research papers were included in this study, with a total of 4,776 patients with endometrial cancer. Non-estrogen-related endometrial carcinoma (NEEC) vs. estrogen-related endometrial carcinoma (EEC) was low in polymerase epsilon (POLE) (OR = 0.49), microsatellite instability (MSI) (OR = 0.45), and copy number low (CNL) (OR = 0.11), while it was high in CNH (OR = 26.76). G3 EEC vs. G1–2 EEC POLE (OR = 1.98), MSI (OR = 1.74), and CNH (OR = 5.57) were high, whereas it was low in CNL (OR = 0.23), low in FIGO II–IV vs. FIGO I in POLE (OR = 0.39) and CNH (OR = 0.64), and high in FIGO II–IV vs. FIGO I in CNH (OR = 3.05). There was no difference in MSI prevalence in FIGO II–IV vs. FIGO I. POLE (OR = 0.64) and CNL (OR = 0.75) were low in myometrial invasion depths ≥50% and lower in myometrial invasion depths <50%, and CNL (OR) was higher in CNH (OR) than in myometrial invasion depths <50%. There was no difference in MSI between different myometrial invasion depths. MSI (OR = 1.69) and CNH (OR = 2.12) were higher in lymphatic vascular infiltration (LVSI) vs. no LVSI; CNL (OR = 0.39) was lower in LVSI than in no LVSI. There was no difference in POLE in the presence or absence of LVSI. Lymph node metastasis with and without lymph node metastasis in POLE (OR = 0.25) and CNL (OR = 0.31) were lower, and CNH (OR = 3.06) was higher in lymph node metastasis than in no lymph node metastasis. There was no difference in MSI in the presence or absence of lymph node metastasis.

**Conclusions:**

POLE patients predominated in pathological features of early-stage endometrial cancer and had better prognosis. MSI patients were more likely to be found in EEC and G3 EEC as well as LVSI. Nearly half of G3 EEC as well as LVSI were present in MSI patients, and CNH patients were more likely to be found to have pathological features of advanced endometrial cancer and poor prognosis, providing evidence that CNH is a high-risk cancer. Patients with CNL were more likely to be found to have pathological features of early-stage endometrial cancer and good prognosis, and CNL was present in large numbers in both early-stage and late-stage endometrial cancers. CNL does not yet have a precise prognostic value.

**Systematic review registration:**

https://www.crd.york.ac.uk/PROSPERO/, identifier CRD42024563661.

## 1 Introduction

Endometrial carcinoma (EC) is the most common gynecologic cancer diagnosed in developed countries and its incidence is increasing (1). Endometrial cancer is more than just a disease; it encompasses several different histologies, the most common being endometrioid carcinoma, followed by serous and clear cell carcinoma. Histologic subtype and other clinicopathologic features such as stage, tumor grade, and presence of lymphovascular space invasion correlate with prognosis, and these variables are used to guide surgery and adjuvant therapy Although many cases are low grade and early diagnosed, a significant number of tumors are diagnosed at a late stage or recur after initial treatment. Even in low-grade and early-stage tumors, a significant percentage will unexpectedly recur. In such cases, the prognosis is poor. Traditional pathology reports of endometrial cancer have limitations in terms of poor reproducibility of tumor types and in the identification of those tumors that unexpectedly recur. Currently, endometrial cancer is typed primarily by morphology, sometimes supplemented by immunohistochemical studies. This inaccurate assessment has led to the conflation of different prognostic subgroups of EC in clinical trials and consequently to misinterpretation of treatment efficacy. The publication of The Cancer Genome Atlas (TCGA) in 2013 on the molecular typing of endometrial carcinoma represented a major step forward in our understanding of the disease (2). TCGA clearly demonstrated the molecular diversity of EC, identifying four distinct molecular subgroups based on somatic copy number alterations and tumor mutational load: the POLE hypermutated phenotype with the best prognosis, the MSI phenotype with an intermediate prognosis, the CNH phenotype with the worst prognosis, and the CNL phenotype with a good to intermediate prognosis. Subsequent studies have shown and validated the prognostic relevance of this molecular stratification, using alternative markers, enabling the identification of four subgroups of EC similar to those described by the TCGA. In 2021, molecular typing has been incorporated into the ESGO–ESTRO–ESP management guidelines (3). As the current management and treatment of EC is mainly based on clinicopathological features, the inclusion of molecular typing will influence the current management stratification and treatment modalities.

Since the description of the four molecular subtypes and the demonstration of their prognostic significance, there has been a significant increase in clinical studies on how to incorporate molecular staging into endometrial cancer. An unresolved issue with the currently proposed molecular stratification of EC is how to classify and manage EC with multiple molecular features, often referred to as “multiple classifiers.” Leon-Castillo et al. (4) reported on tumors with more than one molecular feature. They found that in a large cohort, 3% of tumors were p53abn but also showed one or more additional molecular features: MMRd, POLEmut, or both. However, some of the data on pathologic features were not statistically significant across studies, and a complete summary is not yet available. These data may be critical in guiding clinical study design and establishing optimal customized management of EC patients. Therefore, in this paper, researchers used statistical methods to analyze the published articles on the subject to observe the variability in the prevalence of the four TCGA subgroups in histologic type, FIGO grading, FIGO staging, LVSI, depth of myometrial invasion, and lymph node status, to explore whether different molecular typing is associated with certain specific pathological features, which could help provide a theoretical basis for stratified management, and researchers hope that by describing the association between molecular typing and pathologic features, patients can be treated appropriately based on the clinicopathologic features or molecular typing risk assessment criteria used. Subgroups for which precise prognoses are not currently available should be studied further to determine the molecular phenotypes that assess their prognosis.

## 2 Methods

### 2.1 Sources of data and search strategies

The primary literature search was conducted by searching the Chinese databases of CNKI, Wanfang Database, and VIP Scientific and Technical Journal Database and the English databases of PubMed, Embase, and Web of Science to obtain relevant diagnostic studies in English and Chinese on the molecular characterization of pathologic features of endometrial cancer from the beginning of the database to May 2024. MeSH and Emtree terms and subject terms were used for primary literature search in all fields (three databases): endometrial neoplasms, MMR, POLE, and p53. No restrictions were used in the search, and the literature was searched using a combination of subject terms and keywords. Citations of the included literature were traced back to broaden the scope of the search. The search strategy is as follows: ((“Endometrial Neoplasms” [Mesh]) OR ((((((((((((((((((((Endometrial Neoplasm [Title/Abstract]) OR (Neoplasm, Endometrial [Title/Abstract])) OR (Neoplasms, Endometrial [Title/Abstract])) OR (Endometrial Carcinoma [Title/Abstract])) OR (Carcinoma, Endometrial [Title/Abstract])) OR (Carcinomas, Endometrial [Title/Abstract])) OR (Endometrial Carcinomas [Title/Abstract])) OR (Endometrial Cancer [Title/Abstract])) OR (Cancer, Endometrial [Title/Abstract])) OR (Cancers, Endometrial [Title/Abstract])) OR (Endometrial Cancers [Title/Abstract])) OR (Endometrium Cancer [Title/Abstract])) OR (Cancer, Endometrium [Title/Abstract])) OR (Cancers, Endometrium [Title/Abstract])) OR (Cancer of the Endometrium [Title/Abstract])) OR (Carcinoma of Endometrium [Title/Abstract])) OR (Endometrium Carcinoma [Title/Abstract])) OR (Endometrium Carcinomas [Title/Abstract])) OR (Cancer of Endometrium [Title/Abstract])) OR (Endometrium Cancers [Title/Abstract]))) AND ((((mismatch repair [Title/Abstract] OR MMR [Title/Abstract] OR microsatellite [Title/Abstract] OR MSI [Title/Abstract] OR hypermutated [Title/Abstract]) AND (POLE [Title/Abstract] OR polymerase-ϵ [Title/Abstract] OR ultramutated [Title/Abstract])) AND (p53 [Title/Abstract] OR TP53 [Title/Abstract] OR copy number [Title/Abstract])) OR (TCGA [Title/Abstract] OR the cancer genome atlas [Title/Abstract] OR ProMisE [Title/Abstract])).

### 2.2 Inclusion and exclusion criteria

#### 2.2.1 Inclusion criteria

1. The study subjects were all patients who were treated with surgery for the first time and had endometrial cancer on postoperative pathology and patients who did not receive treatment such as radiotherapy or endocrine therapy before surgery. There was no restriction on race, nationality, or age.
2. The type of study is a diagnostic study of pathological features related to the molecular characteristics of endometrial cancer from the time of bank-in until May 2024.
3. The outcome indicators were histologic type [estrogen-related endometrial carcinoma (EEC) and non-estrogen-related endometrial carcinoma (NEEC)], FIGO classification (FIGO stage I and FIGO stages II–IV), FIGO stage (G1–2 and G3), LVSI, depth of myometrial invasion, and lymph node status in each TCGA group.
4. Literature was published in full text in both English and Chinese.

#### 2.2.2 Exclusion criteria

The exclusion criteria were as follows:

1. sample size <10;
2. any type of study, such as case reports, case reports, letters, and comments, from which accurate or complete raw data cannot be extracted or from which the data are limited to a single patient;
3. duplicate data or studies from the same center, excluding duplicate cases;
4. multiple reports from a single center, selecting the literature with the largest sample size without duplicates;
5. incomplete TCGA classification (i.e., not all TCGA subgroups were surveyed);
6. lack of data in the literature that incorporates pathological features; and
7. reviews, literature laboratory studies, or animal experiments.

### 2.3 Data extraction from research literature

Initial screening of the literature retrieved from the database was conducted independently by two researchers to exclude articles that did not match the type of study and did not have the appropriate indicators based on reading the titles and abstracts. After the initial screening, the articles were read carefully in their entirety to evaluate the quality of literature in the final included articles. Relevant information was extracted in the final included literature, and the data were extracted from the original study without modification. The main data extracted were the number of prevalent and total number of patients with histologic type, FIGO grade, FIGO stage, LVSI, depth of myometrial invasion, and lymph node status in each TCGA group. Further extracted data included country, time of enrollment, method of patient selection, review of pathologic diagnosis, molecular/immunohistochemical methods used to assign ECs to specific TCGA groups, and patients potentially excluded from molecular/immunohistochemical analyses. Results from the two investigators were compared, and any discrepancies or disagreements were resolved by discussion with a third investigator, who evaluated the same data. The study authors were contacted for additional information to determine the final literature to be included, if necessary.

### 2.4 Literature quality assessment

The methodological quality of the included literature was assessed by the researchers according to the Cochrane Assessment Tool, and quality evaluation of the included diagnostic studies was based on Cochrane’s QUADAS-2 scale. We assessed the risk of bias in the studies in four areas: patient selection (were patients selected consecutively and/or were inclusion criteria and enrollment period specified)?, index tests (were immunohistochemical/molecular analyses unbiased)?, reference standard (were histologic sections reviewed to confirm pathologic diagnosis)?, and flow (were immunohistochemical/molecular analyses performed in at least 90% of the included patients)?. The results were entered into the Review Manager (RevMan) 5.4.1 software to produce diagnostic study quality assessment charts and risk of bias charts.

### 2.5 Statistical analysis

Meta-analysis of the data was done using Review Manager (RevMan) 5.4.1 software. The chi-square test and *I* ^2^ statistic were used to test the heterogeneity of the data. *I* ^2^ statistic is an important indicator of heterogeneity. Values of 25%, 50%, and 75% represent low, medium, and high heterogeneity, respectively. If *P >*0.1 and *I* ^2^ ≤50%, the study is considered homogeneous, and meta-analysis was performed using the fixed-effects model. If *P <*0.1 and *I* ^2^ >50%, the study is considered heterogeneous, and meta-analysis was performed using the random-effects model. In this study, the odds ratio (OR) was used as the effect size, and 95% confidence interval (95% CI) was calculated to study the statistical correlation between endometrial cancer in each subgroup of TCGA and histological type, tumor grade, FIGO stage, LVSI, depth of myometrial invasion, and lymph node status (*P* < 0.05 was statistically significant). The researchers used the Stata 14.0 software to combine the effect sizes for each included data using one-by-one exclusion of each included study before combining the effect sizes, and examined the robustness of the results and the high heterogeneity of the results by observing the changes in ORs and 95% CIs of the combined results. At the same time, the researchers tested the publication bias of the included literature by making a funnel plot of the forest plots with the horizontal coordinate as the OR value and the vertical coordinate as the standard error SE (log[OR]) after the inclusion of more than 10 literature and passing the sensitivity test. A small sample size and low research precision were distributed at the bottom of the funnel plot and dispersed around; a large sample size and high research precision were distributed at the top of the funnel plot and concentrated toward the middle.

## 3 Results

### 3.1 Results of the literature search

**Figure 1:**
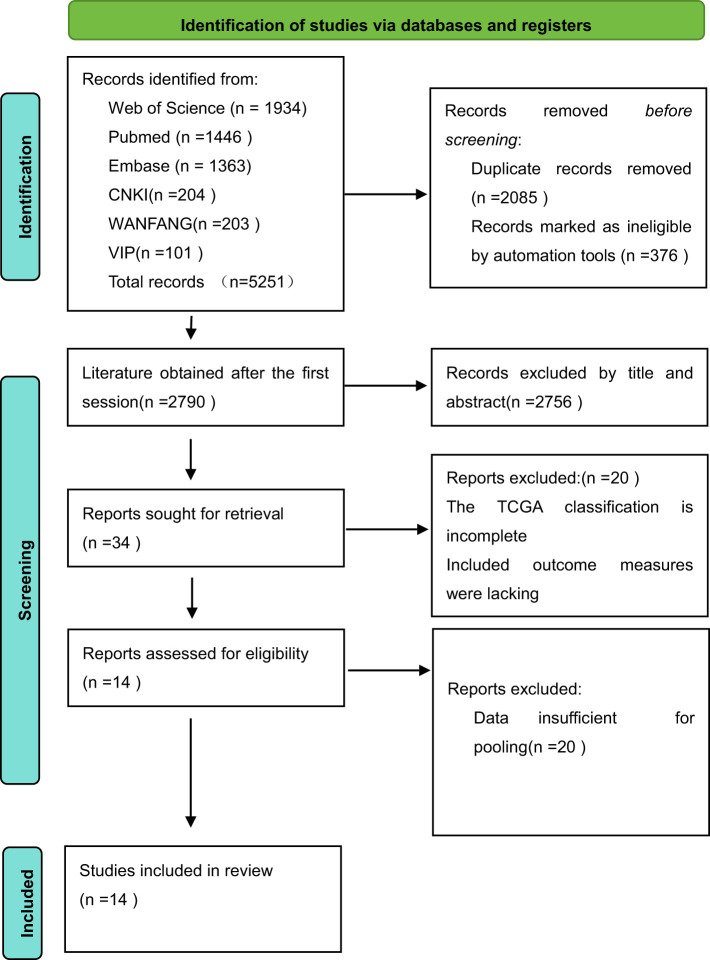
The RevMan search flowchart.

This study initially retrieved 5,251 studies from the literature, with 508 studies in Chinese and 4,743 studies in English. There were 2,790 studies that were obtained by utilizing EndNote to eliminate duplicates as well as unqualified literature, 34 studies that were obtained by reading the titles and abstracts, 2,756 studies that were eliminated, and 20 studies that were obtained by further reading the full text. Finally, total of 14 studies were included in the review, 12 of which were retrospective studies and 2 were prospective studies. The detailed search process is shown in **Figure 1**.

### 3.2 Basic characteristics of all included studies

Eleven of the included publications were in English and three were in Chinese, with 4,776 patients having endometrial cancer. The original studies of the TCGA used molecular analyses to assess molecular subgroups. One study assessed the POLE subgroup by sequencing and the other subgroups by NGS, whereas the other studies assessed the POLE subgroup by sequencing and the other subgroups by immunohistochemistry. Twelve publications studied the relationship between the four molecular typing assays and histologic type of endometrial cancer patients, 14 papers examined the relationship between the four molecular typing assays and tumor grading of endometrial cancer patients, 8 papers examined the relationship between the four molecular typing assays and FIGO staging of endometrial cancer patients, 11 papers examined the relationship between the four molecular typing assays and LVSI of endometrial cancer patients, 8 papers investigated the relationship between the four molecular staging categories and myometrial invasion in patients with endometrial cancer, and 5 papers investigated the relationship between the four molecular staging categories and lymph node metastasis in patients with endometrial cancer. The basic characteristics of the included literature are detailed in **Table 1**.

**Table 1:** Basic characteristics of the included literature.

Author	Year	Country	Type of study	Duration of study	Number of persons included	Molecular typing assays			
						POLE	MSI	CNH	CNL
Kandoth C (TCGA) (1)	2013	America	Retro	Unclear	373	Mol	Mol	Mol	Mol
Talhouk (5)	2015	Canada	Retro	2002–2009	143	Mol	IHC	IHC	IHC
Stelloo (6)	2016	Holland	Retro	1990–1997; 2001–2006	834	Mol	IHC	IHC	IHC
Talhouk (7)	2017	Canada	Retro	1983–2013	319	Mol	IHC	IHC	IHC
Cosgrove (8)	2018	America	Retro	2003–2007	982	Mol	IHC	IHC	IHC
Kommoss (9)	2018	Germany	Retro	2003–2013	452	Mol	IHC	IHC	IHC
Kolehmainen (10)	2020	Finland	Retro	2007–2012	604	Mol	IHC	IHC	IHC
Timmerman (11)	2020	Belgium	Pro	2017–2019	108	Mol	IHC	IHC	IHC
Eriksson (12)	2021	Sweden	Pro	2011–2015	339	Mol	IHC	IHC	IHC
Huvila (13)	2021	Finland	Retro	2008–2018	60	Mol	NGS	NGS	NGS
Devereaux (14)	2021	America	Retro	2019–2021	310	Mol	IHC	IHC	IHC
Zhao Luyang (15)	2021	China	Retro	2018–2021	66	Mol	IHC	IHC	IHC
Li Wengqi (16)	2021	China	Retro	2020–2021	100	Mol	IHC	IHC	IHC
Sun Lili (17)	2022	China	Retro	2015–2017; 2017–2022	86	Mol	IHC	IHC	IHC

Retro, retrospective study; Pro, prospective study; Mol, molecular assay; IHC, immunohistochemical assay; NGS, next-generation sequencing.

### 3.3 Evaluation of the quality of the literature

**Figure 2:**
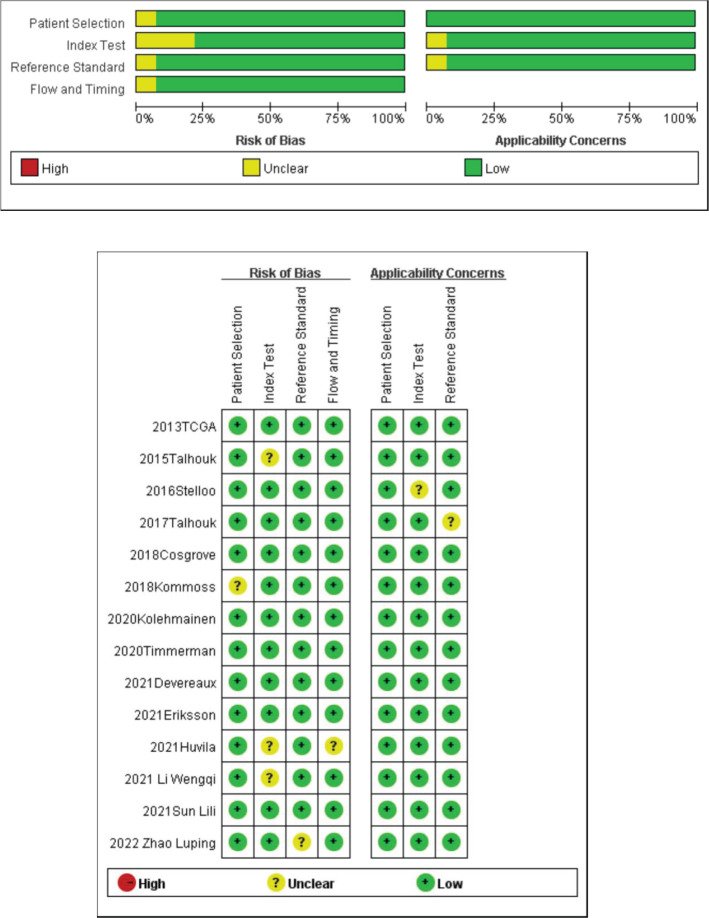
Quality evaluation of the included literature and risk of bias map.

The quality evaluation of 17 papers was conducted using the evaluation methods recommended by the Cochrane Evaluation Handbook 5.1.0. According to the QUADAS-2 scale, we evaluated the quality of the included papers and assessed the risk of bias in four aspects (patient selection, index test, reference standard, and flow rate), and the quality evaluation diagram is shown in **Figure 2**. The quality of the included diagnostic studies was better, and the risk of bias was low.

### 3.4 Meta-analysis results

#### 3.4.1 Relationship between molecular typing and histologic type of endometrial cancer (NEEC vs. EEC)

A total of 12 studies extracted the values of NEEC and EEC for comparison, and a total of 2,817 patients were included in these 12 studies, including a total of 520 patients with NEEC and 2,297 patients with EEC. Of all the NEEC patients included, there were a total of 20 cases (3.85%) in the POLE subgroup, 95 cases (18.27%) in the MSI subgroup, 352 cases (67.69%) in the CNH subgroup, and 53 cases (10.19%) in the CNL subgroup. Of all the EEC patients included, there were a total of 180 cases (7.84%) in the POLE subgroup, 816 cases (33.52%) in the MSI subgroup, 148 cases (6.44%) in the CNH subgroup, and 1,153 cases (50.20%) in the CNL subgroup. The results are shown in **Table 2**.

**Table 2:** Prevalence of molecular typing in different pathological features.

Pathological category	Total	POLE mutant type	MSI type	CNH type	CNL type
Histological type	2,817				
EEC	2,297	180 (7.84%)	816 (33.52%)	148 (6.44%)	1,153 (50.20%)
NEEC	520	20 (3.85%)	95 (18.27%)	352 (67.69%)	53 (10.19%)
Pathological grading of EEC	4,062				
G1–2	3,363	189 (5.62%)	1,066 (31.70%)	158 (4.70%)	1,950 (57.98%)
G3	699	76 (10.87%)	300 (42.92%)	148 (21.17%)	175 (25.04%)
FIGO instalment	3,220				
I	2,389	172 (7.20%)	825 (34.53%)	248 (10.38%)	1,144 (47.89%)
II–IV	831	23(2.77%)	291 (35.02%)	211 (25.39%)	306 (36.82%)
Depth of myometrial invasion	2,360				
<50%	1,420	86 (6.06%)	504 (35.49%)	166 (11.69%)	664 (46.76%)
≥50%	940	39 (4.15%)	356 (38.87%)	177 (18.83%)	368 (39.19%)
LVSI	3,899				
Negative	3,032	177 (5.84%)	910 (30.01%)	319 (10.52%)	1,626 (53.63%)
Positive	867	48 (5.54%)	365 (42.10%)	194 (22.38%)	260 (30.00%)
Lymph node metastasis	905				
Negative	840	86 (10.24%)	241 (28.69%)	164 (19.52%)	348 (41.43%)
Positive	105	0 (0.00%)	30 (28.57%)	46 (43.81%)	28 (26.67%)

**Figure 3:**
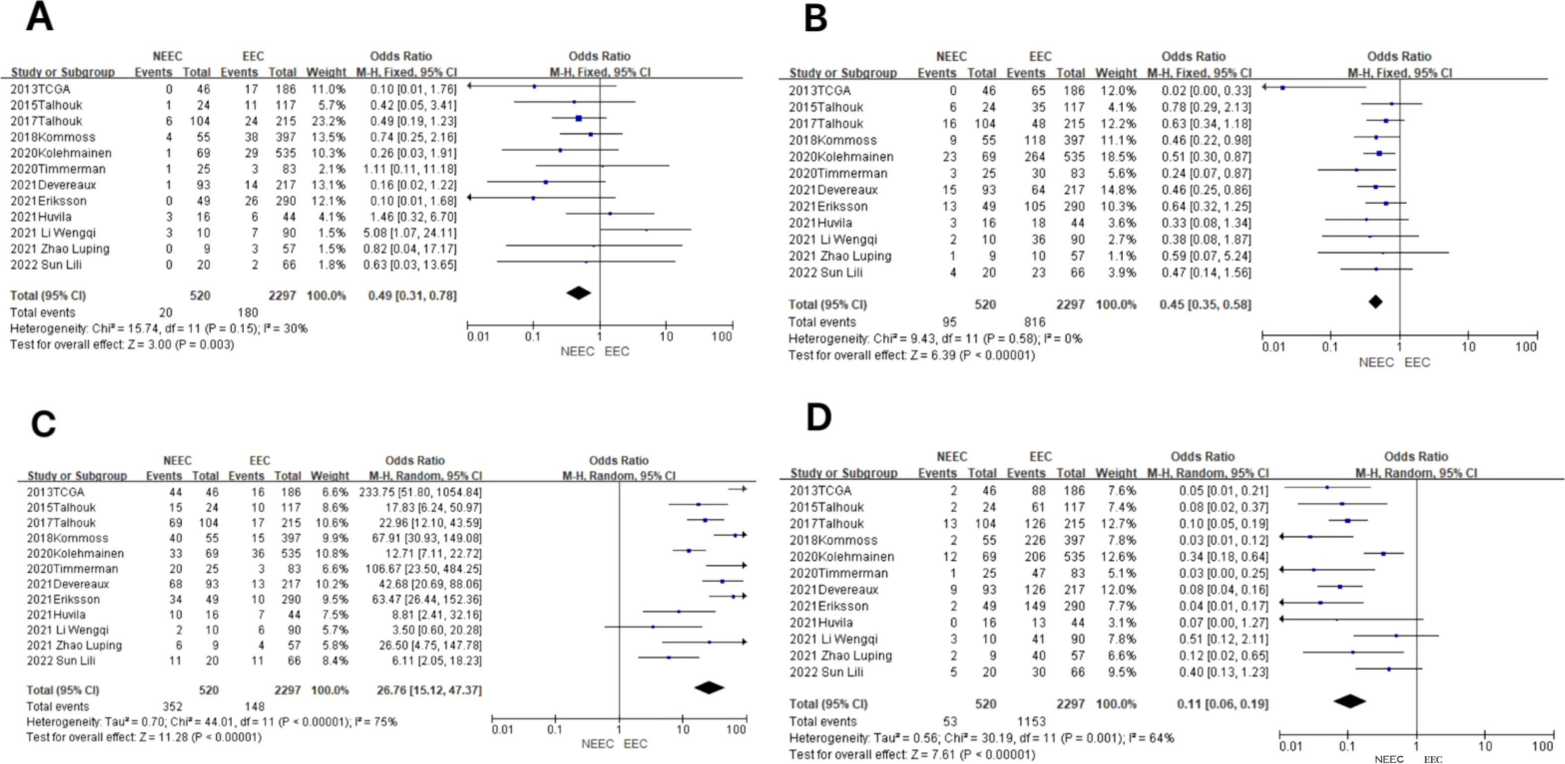
Relationship between molecular typing and histological types of endometrial carcinoma. **(A)** The POLE subgroup, **(B)** the MSI subgroup, **(C)** the CNH subgroup, and **(D)** the CNL subgroup.

In the POLE subgroup, the heterogeneity test showed that there was homogeneity of results (*I* ^2^ = 30%, *P* = 0.15). The results showed that between NEEC and EEC patients (OR = 0.49, 95% CI 0.31–0.78, *P* = 0.003), after observing the combined effect sizes, the diamond shape did not intersect with the null line, indicating that there was a low prevalence of the POLE subgroup in NEEC patients as compared to EEC and the difference was statistically significant. The results are shown in **Figure 3A**.

In the MSI subgroup, the heterogeneity test showed that there was homogeneity of results (*I* ^2^ = 0%, *P* = 0.58), so meta-analysis was performed using a fixed-effects model. The results showed that in the MSI subgroup, the diamond shape between NEEC and EEC patients (OR = 0.45, 95% CI 0.35–0.58, *P* < 0.00001) and after observing the combined effect sizes did not intersect with the null line, which indicated that there was a low prevalence of the MSI subgroup among NEEC patients compared to EEC, and the results were statistically significant. The results are shown in **Figure 3B**.

In the CNH subgroup, the heterogeneity test showed a large heterogeneity of results (*I* ^2^ = 75%, *P* < 0.00001), and no significant improvement in heterogeneity was found by the one-by-one exclusion method, so meta-analysis was performed using a random-effects model. The results showed that among the CNH subgroups, the prevalence of the CNH subgroup was high among NEEC and EEC patients (OR = 26.76, 95% CI 15.12–47.37, *P* < 0.00001), and the diamond shape after observing the combined effect sizes did not intersect with the null line, which indicated a high prevalence of the CNH subgroup among NEEC patients compared to EEC and that the difference was statistically significant. The results are shown in **Figure 3C**.

In the CNL subgroup, the heterogeneity test showed moderate heterogeneity of results (*I* ^2^ = 64%, *P* = 0.001), so meta-analysis was performed using the random-effects model. The results showed that in the CNL subgroup, between NEEC and EEC patients (OR = 0.11, 95% CI 0.06–0.19, *P* < 0.00001), the diamond shape after observing the combined effect sizes did not intersect with the null line, which indicated that the prevalence of the CNL subgroup was low and the difference was statistically significant in the NEEC compared to the EEC patients. The results are shown in **Figure 3D**.

#### 3.4.2 Relationship between molecular typing and pathologic grading of endometrioid carcinoma (G3 vs. G1–2)

The values of endometrioid carcinoma G3 and G1–2 were extracted for comparison in the 14 included studies, which included a total of 4,062 patients, of which there were a total of 699 G3 patients and 3,363 G1–2 patients. Among all G3 patients included, there were a total of 76 cases (10.87%) in the POLE subgroup, 300 cases (42.92%) in the MSI subgroup, 148 cases (21.17%) in the CNH subgroup, and 175 cases (25.04%) in the CNL subgroup. Among all G1–2 patients included, there were a total of 189 cases (5.62%) in the POLE subgroup, a total of 1,066 cases (31.70%) in the MSI subgroup, a total of 158 cases (4.70%) in the CNH subgroup, and a total of 1,950 cases (57.98%) in the CNL subgroup. The results are shown in **Table 2**.

**Figure 4:**
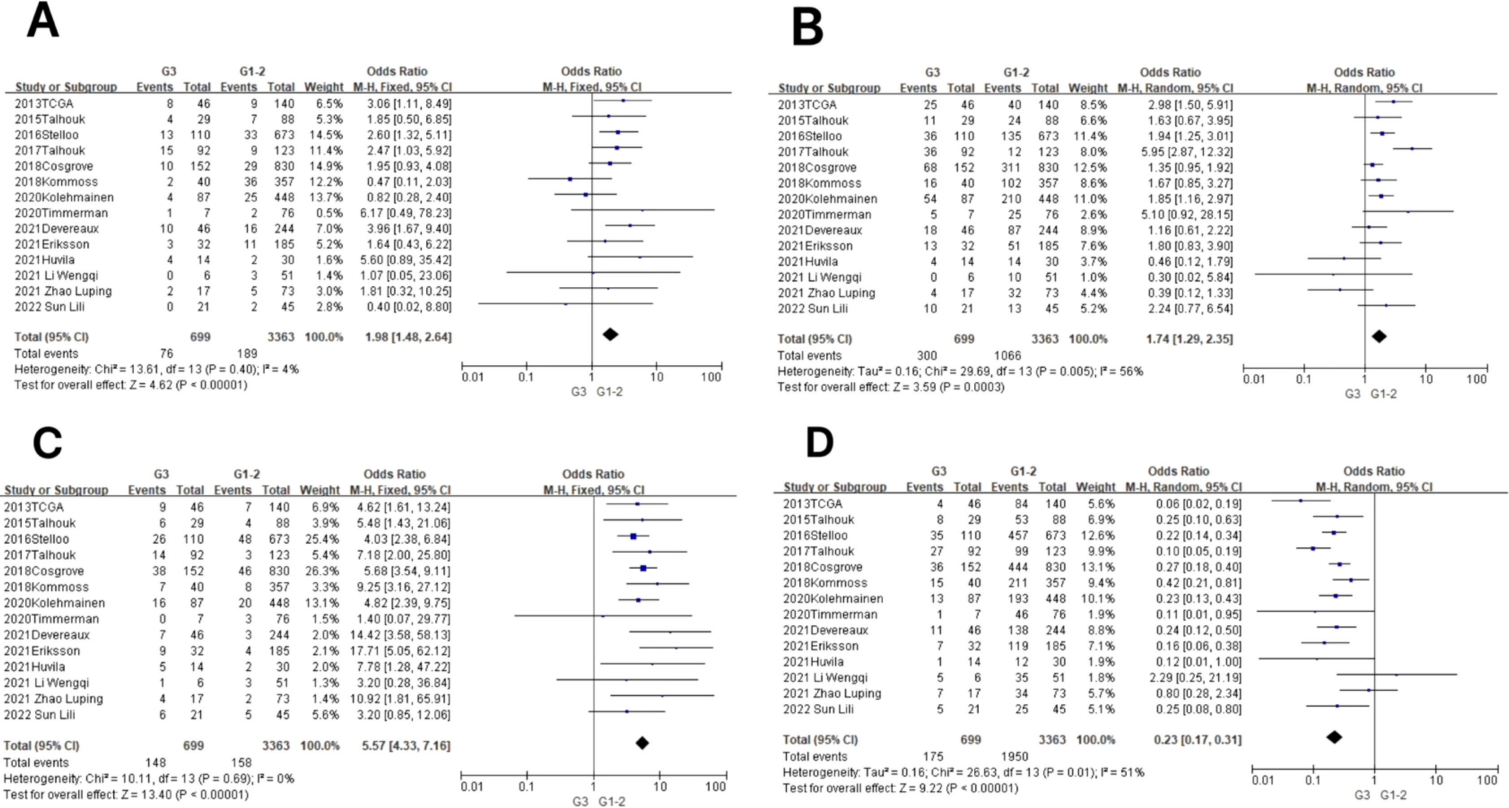
Relationship between molecular typing and pathological grading of endometrioid carcinoma. **(A)** The POLE subgroup, **(B)** the MSI subgroup, **(C)** the CNH subgroup, and **(D)** the CNL subgroup.

In the POLE subgroup, the heterogeneity test showed that there was homogeneity of results (*I* ^2^ = 4%, *P* = 0.40), so meta-analysis was performed using the fixed-effects model. The results showed that in the POLE subgroup, between G3 and G1–2 patients (OR = 1.98, 95% CI 1.48–2.64, *P* < 0.00001), the diamond shape after observing the combined effect sizes did not intersect with the null line, which indicated that the prevalence of the POLE subgroup was high in G3 EEC patients compared to G1–2 EEC patients, and the difference was statistically significant. The results are shown in **Figure 4A**.

In the MSI subgroup, the heterogeneity test showed moderate heterogeneity of results (*I* ^2^ = 56%, *P* = 0.005), so meta-analysis was performed using the random-effects model. The results showed that in the MSI subgroup, between G3 and G1–2 patients (OR = 1.74, 95% CI 1.29–2.35, *P* = 0.0003), the rhombus after observing the combined effect sizes did not intersect with the null line, which indicated that there was a high prevalence of the MSI subgroup in patients with G3 EEC as compared to patients with G1–2 EEC, and the results were statistically significant. The results are shown in **Figure 4B**.

In the CNH subgroup, the heterogeneity test showed that there was homogeneity of results (*I* ^2^ = 0%, *P* = 0.69), so meta-analysis was performed using the fixed-effects model. The results showed that among the CNH subgroups, the prevalence of CNH subgroups was high among G3 vs. G1–2 patients (OR = 5.57, 95% CI 4.33–7.16, *P* < 0.00001), and the diamond shape after observing the combined effect sizes did not intersect with the null line, indicating that there was a high prevalence of CNH subgroups among the G3 EEC patients as compared to the G1–2 EEC patients and that the difference was statistically significant. The results are shown in **Figure 4C**.

In the CNL subgroup, the heterogeneity test showed moderate heterogeneity of results (*I* ^2^ = 51%, *P* = 0.01), so meta-analysis was performed using the random-effects model. The results showed that among the CNL subgroups, between G3 and G1–2 patients (OR = 0.23, 95% CI 0.17–0.31, *P* < 0.00001), the diamond shape after observing the combined effect sizes did not intersect with the null line, indicating that there was a low prevalence of CNL subgroups among G3 EEC patients compared to G1–2 EEC patients and that the difference was statistically significant. The results are shown in **Figure 4D**.

#### 3.4.3 Relationship between molecular typing and FIGO staging of endometrial cancer (2009) (FIGO stages II–IV vs. FIGO stage I)

A total of eight studies extracted the values of endometrial cancer FIGO stages II–IV compared to FIGO stage I. A total of 3,220 patients were included, of which a total of 831 patients were in FIGO stages II–IV and a total of 2,389 patients were in FIGO stage I. The total number of patients in the FIGO stage II–IV subgroup was 831 (2.02%), and in the CNH subgroup, it was 291 (2.02%). Of all FIGO stage II–IV patients included, there were a total of 23 cases (2.77%) in the POLE subgroup, 291 cases (35.02%) in the MSI subgroup, 211 cases (25.39%) in the CNH subgroup, and 306 cases (36.82%) in the CNL subgroup. Of all FIGO stage I patients included, there were a total of 172 cases in the POLE subgroup (7.20%), a total of 825 cases (34.53%) in the MSI subgroup, a total of 248 cases (10.38%) in the CNH subgroup, and a total of 1,144 cases (47.89%) in the CNL subgroup. The results are shown in **Table 2**.

**Figure 5:**
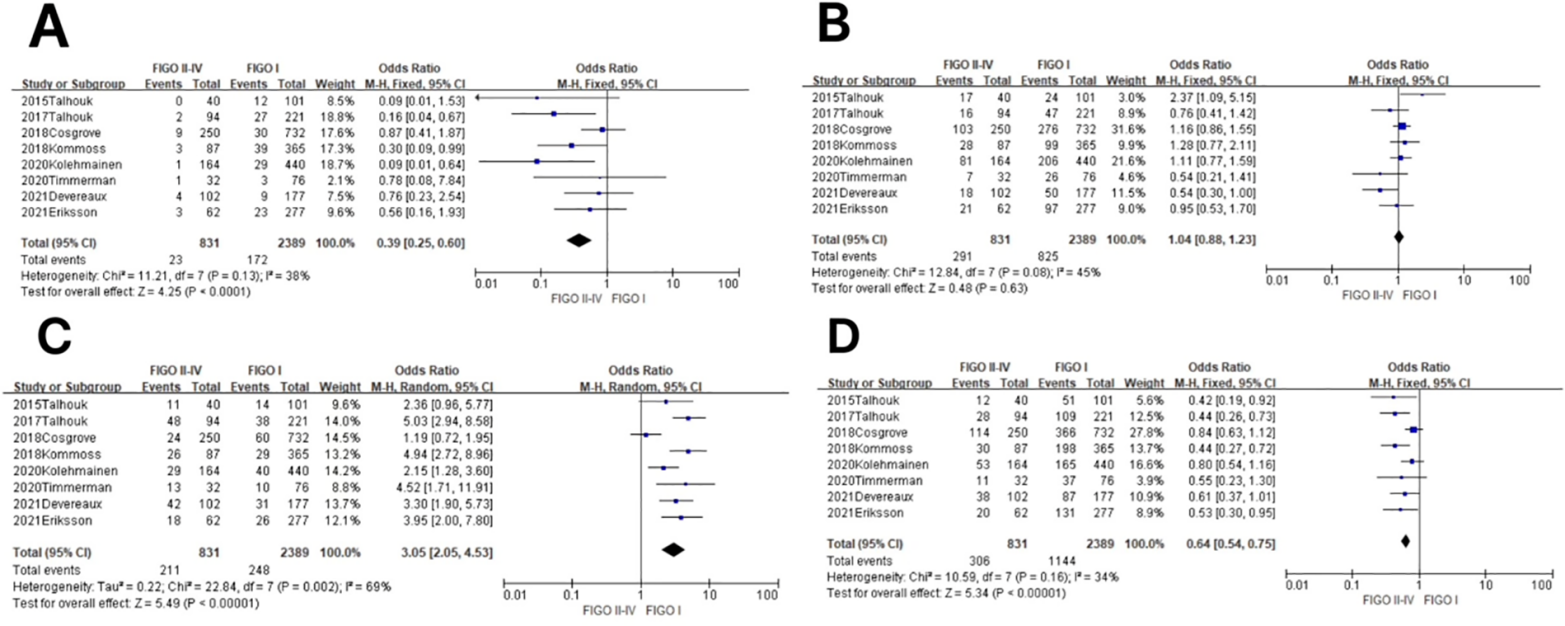
Relationship between molecular staging and FIGO (2009) staging of endometrial cancer. **(A)** The POLE subgroup, **(B)** the MSI subgroup, **(C)** the CNH subgroup, and **(D)** the CNL subgroup.

In the POLE subgroup, the heterogeneity test showed that there was homogeneity of results (*I* ^2^ = 38%, *P* = 0.13), so meta-analysis was performed using the fixed-effects model. The results showed that in the POLE subgroup, between FIGO stage II–IV and FIGO stage I patients (OR = 0.39, 95% CI 0.25–0.60, *P* < 0.0001), the diamond shape after observing the combined effect sizes did not intersect with the null line, which indicated that the prevalence of the POLE subgroup was low in FIGO stage II–IV patients compared to FIGO stage I, and the difference was statistically significant. The results are shown in **Figure 5A**.

In the MSI subgroup, the heterogeneity test showed that there was homogeneity of results (*I* ^2^ = 45%, *P* = 0.08), so meta-analysis was performed using the fixed-effects model. The results showed that in the MSI subgroup, between FIGO stage II–IV and FIGO stage I patients (OR = 1.04, 95% CI 0.88–1.23, *P* = 0.63), the diamond shape after observing the combined effect sizes intersected with the null line, indicating that the results were not statistically significant. The results are shown in **Figure 5B**.

In the CNH subgroup, the heterogeneity test showed moderate heterogeneity of results (*I* ^2^ = 69%, *P* = 0.002), so meta-analysis was performed using the random-effects model. The results showed that in the CNH subgroup, between FIGO stage II–IV and FIGO stage I patients (OR = 3.05, 95% CI 2.05–4.53, *P* < 0.00001), the diamond shape after observing the combined effect sizes did not intersect with the null line, which indicated that the prevalence of the CNH subgroup was high in patients with FIGO stages II–IV compared to patients with FIGO stage I, and the difference was statistically significant. The results are shown in **Figure 5C**.

In the CNL subgroup, the heterogeneity test showed that there was a large heterogeneity of results (*I* ^2^ = 34%, *P* = 0.16), so meta-analysis was performed using the fixed-effects model. The results showed that in the CNL subgroup, between FIGO stage II–IV and FIGO stage I patients (OR = 0.64, 95% CI 0.54–0.75, *P* < 0.00001), the diamond shape after observing the combined effect sizes did not intersect with the null line, which indicated that the prevalence of the CNL subgroup was low among FIGO stage II–IV patients compared to FIGO stage I patients, and the difference was statistically significant. The results are shown in **Figure 5D**.

#### 3.4.4 Relationship between molecular typing and depth of myometrial invasion in endometrial cancer (invasion depth ≥ 50% vs. invasion depth < 50%)

A total of seven studies extracted values for endometrial cancer myometrial invasion depth ≥50% compared with myometrial invasion depth <50% and included a total of 2,360 patients, of which there were a total of 940 patients with myometrial invasion depth ≥50% and a total of 1,420 patients with myometrial invasion depth <50%. Of all patients included with a myometrial invasion depth ≥50%, there were a total of 39 (4.15%) in the POLE subgroup, 356 (38.87%) in the MSI subgroup, 177 (18.83%) in the CNH subgroup, and 368 (39.19%) in the CNL subgroup. Of all patients included with a myometrial invasion depth <50%, there were a total of 86 in the POLE subgroup (6.06%), a total of 504 cases (35.49%) in the MSI subgroup, a total of 166 cases (11.69%) in the CNH subgroup, and a total of 664 cases (46.76%) in the CNL subgroup. The results are shown in **Table 2**.

**Figure 6:**
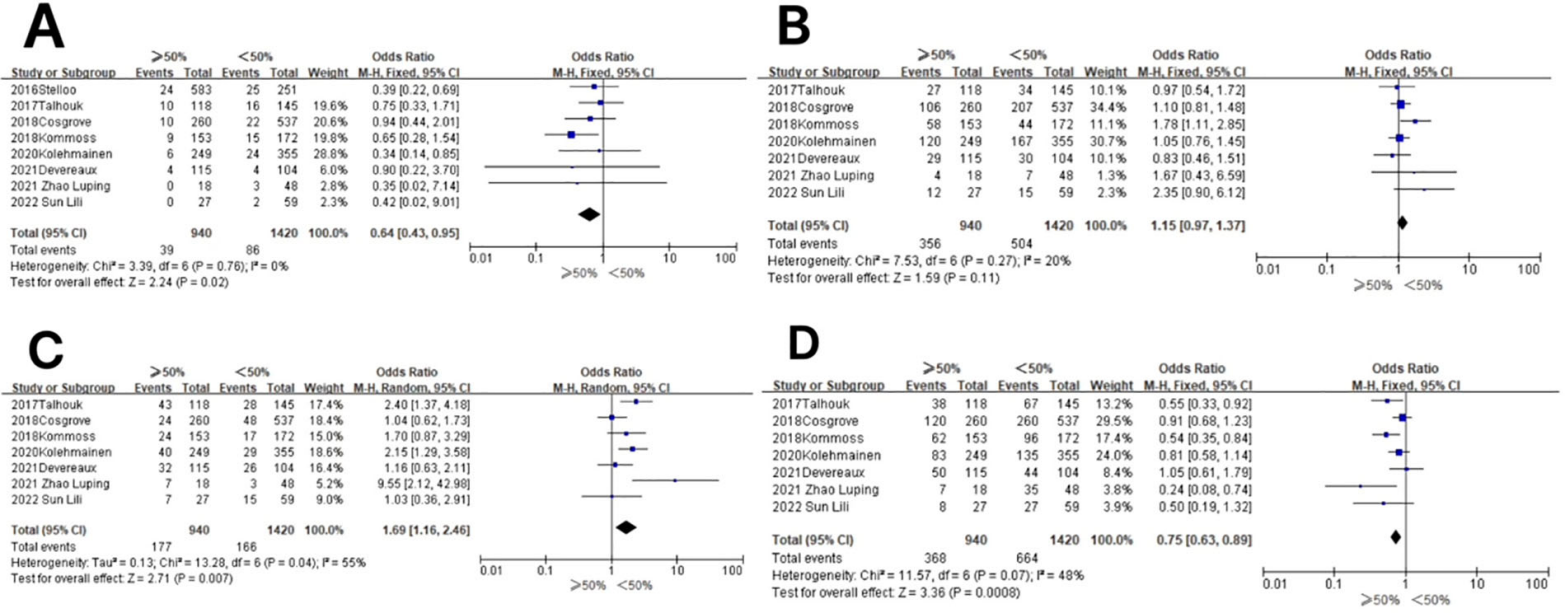
Relationship between molecular typing and depth of myometrial invasion in endometrial carcinoma. **(A)** The POLE subgroup, **(B)** the MSI subgroup, **(C)** the CNH subgroup, and **(D)** the CNL subgroup.

In the POLE subgroup, the heterogeneity test showed that there was homogeneity of results (*I* ^2^ = 0%, *P* = 0.76), so meta-analysis was performed using the fixed-effects model. The results showed that between patients with myometrial invasion depth ≥50% and patients with myometrial invasion depth <50% (OR = 0.64, 95% CI 0.43–0.95, *P* = 0.02), the diamond shape after observing the combined effect sizes did not intersect with the null line, indicating that the prevalence of the POLE subgroup was low in patients with myometrial invasion depth ≥50% compared to those with myometrial invasion depth <50% and that the difference was statistically significant. The results are shown in **Figure 6A**.

In the MSI subgroup, the heterogeneity test showed that there was homogeneity of results (*I* ^2^ = 20%, *P* = 0.27), and therefore, meta-analysis was performed using the fixed-effects model. The results showed that in the MSI subgroup, between patients with myometrial invasion depth ≥50% and patients with myometrial invasion depth <50% (OR = 1.15, 95% CI 0.97–1.37, *P* = 0.11), the diamond shape after observing the combined effect sizes intersected with the null line, which indicated that there was no statistical significance in the results. The results are shown in **Figure 6B**.

In the CNH subgroup, the heterogeneity test showed that there was moderate heterogeneity of results (*I* ^2^ = 55%, *P* = 0.04), so meta-analysis was performed using the random-effects model. The results showed that in the CNH subgroup, the prevalence of CNH was high in the CNH subgroup among patients with myometrial invasion depth ≥50% vs. those with myometrial invasion depth <50% (OR = 1.69, 95% CI 1.16–2.46%, *P* = 0.007), and the diamond shape after observing the combined effect sizes did not intersect with the null line, indicating that the prevalence of CNH was high in the CNH subgroup among patients with myometrial invasion depth ≥50% compared to those with myometrial invasion depth <50%, and the difference was statistically significant. The results are shown in **Figure 6C**.

In the CNL subgroup, the heterogeneity test showed that there was homogeneity of results (*I* ^2^ = 48%, *P* = 0.07), and therefore, meta-analysis was performed using the fixed-effects model. The results showed that among the CNL subgroups, the prevalence of CNL subgroups was low among patients with myometrial invasion depth ≥50% vs. those with myometrial invasion depth <50% (OR = 0.75, 95% CI 0.63–0.89, *P* = 0.0008), and the rhombus shape after observing the combined effect sizes did not intersect with the null line, which indicated that the prevalence of the CNL subgroups was lower in patients with myometrial invasion depth ≥50% compared to those with myometrial invasion depth <50%, and the difference was statistically significant. The results are shown in **Figure 6D**.

#### 3.4.5 Association between molecular typing and lymphovascular infiltration in endometrial cancer (positive vs. negative)

A total of 11 studies extracted the values of lymphovascular infiltration (positive vs. negative) in endometrial cancer for comparison and included a total of 3,899 patients, of which a total of 867 patients had lymphovascular infiltration and a total of 3,032 patients had no lymphovascular infiltration. Of all patients included with lymphovascular infiltration, there were a total of 48 (5.54%) in the POLE subgroup, 365 (42.10%) in the MSI subgroup, 194 (22.38%) in the CNH subgroup, and 260 (30.00%) in the CNL subgroup. Of all patients included without lymphovascular infiltration, there were a total of 177 (5.84%) in the POLE subgroup, a total of 910 cases (30.01%) in the MSI subgroup, a total of 319 cases (10.52%) in the CNH subgroup, and a total of 1,626 cases (53.63%) in the CNL subgroup. The results are shown in **Table 2**.

**Figure 7:**
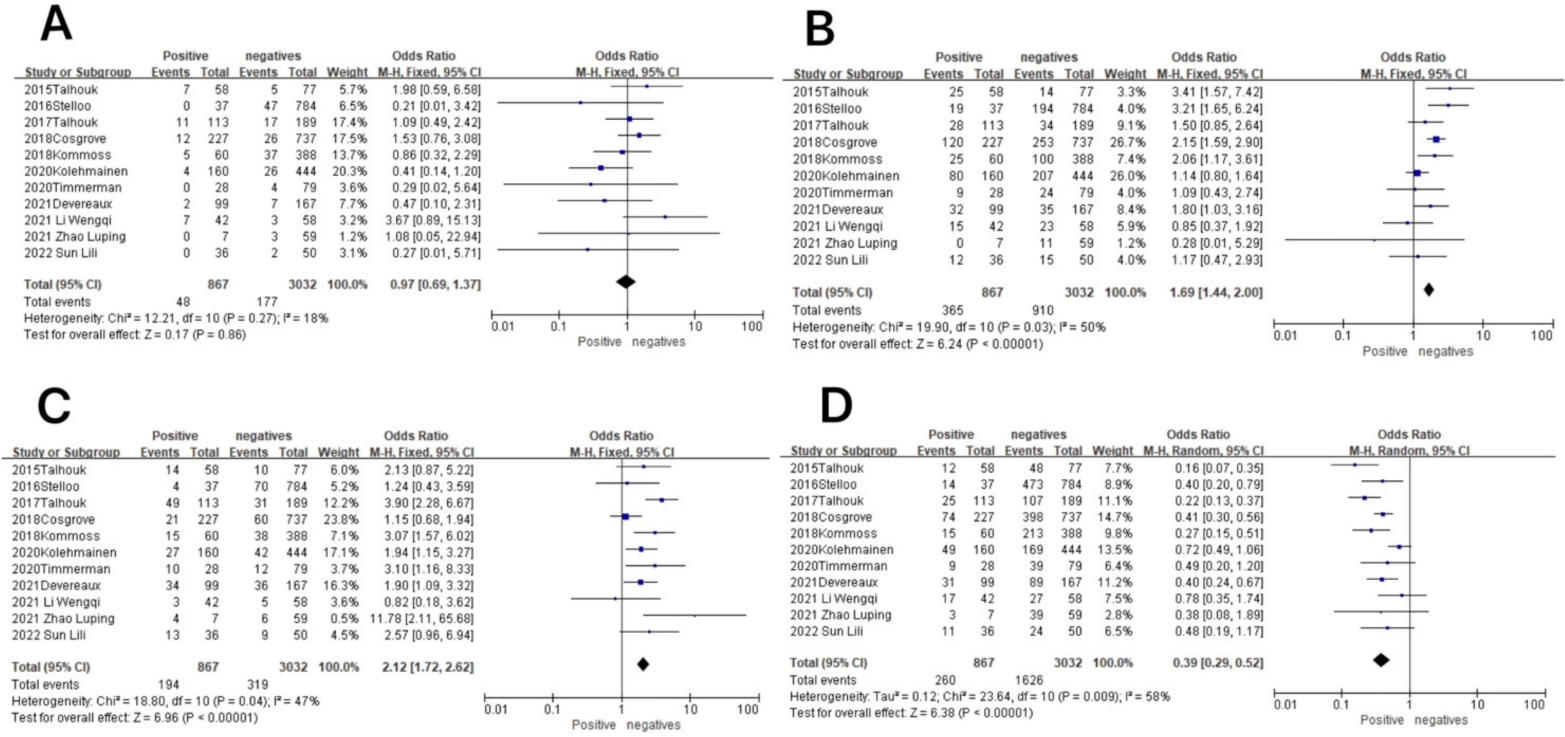
Association between molecular typing and lymphovascular infiltration in endometrial cancer. **(A)** The POLE subgroup, **(B)** the MSI subgroup, **(C)** the CNH subgroup, and **(D)** the CNL subgroup.

In the POLE subgroup, the heterogeneity test showed that there was homogeneity of results (*I* ^2^ = 18%, *P* = 0.27), so meta-analysis was performed using the fixed-effects model. The results showed that in the POLE subgroup, between patients with lymphovascular infiltration and those without lymphovascular infiltration (OR = 0.97, 95% CI 0.69–1.37, *P* = 0.86), the diamond shape after combining the effect sizes intersected with the null line, indicating that the difference was not statistically significant. The results are shown in **Figure 7A**.

In the MSI subgroup, the heterogeneity test showed that there was homogeneity of results (*I* ^2^ = 50%, *P* = 0.03), so meta-analysis was performed using the fixed-effects model. The results showed statistically significant results in the MSI subgroup between patients with lymphovascular infiltration and those without lymphovascular infiltration (OR = 1.69, 95% CI 1.44–2.00, *P* < 0.00001), and the diamond shape after observing the combined effect sizes did not intersect with the null line, which indicated that there was a high prevalence of the MSI subgroup in patients with lymphovascular infiltration as compared to those without lymphovascular infiltration. The results are shown in **Figure 7B**.

In the CNH subgroup, the heterogeneity test showed that there was homogeneity of results (*I* ^2^ = 47%, *P* = 0.04), so meta-analysis was performed using the fixed-effects model. The results showed that among the CNH subgroup, between lymphovascular infiltration and no lymphovascular infiltration patients (OR = 2.12, 95% CI 1.72–2.62, *P* < 0.00001), the diamond shape after observing the combined effect sizes did not intersect with the null line, which indicated that the prevalence of the CNH subgroup was high in lymphovascular infiltration patients as compared to patients with no lymphovascular infiltration and that the difference was statistically significant. The results are shown in **Figure 7C**.

In the CNL subgroup, the heterogeneity test showed moderate heterogeneity of results (*I* ^2^ = 58%, *P* = 0.009), so meta-analysis was performed using the random-effects model. The results showed that in the CNL subgroup, between patients with lymphovascular infiltration and those without lymphovascular infiltration (OR = 0.39, 95% CI 0.29–0.52, *P* < 0.00001), the diamond shape after observing the combined effect sizes did not intersect with the null line, indicating that the prevalence of the CNL subgroup was low in patients with lymphovascular infiltration compared to those without lymphovascular infiltration, and the difference was statistically significant. The results are shown in **Figure 7D**.

#### 3.4.6 Association between molecular typing and lymph node metastatic status of endometrial cancer (positive vs. negative)

A total of six studies extracted values for endometrial cancer lymph node metastasis status (positive vs. negative) for comparison and included a total of 943 patients, of which there were a total of 104 patients with lymph node metastasis and a total of 839 patients without lymph node metastasis. Among all patients included with lymph node metastasis, there were a total of 0 cases (0.00%) in the POLE subgroup, 30 cases (28.57%) in the MSI subgroup, 46 cases (43.81%) in the CNH subgroup, and 28 cases (26.67%) in the CNL subgroup. Among all patients included without lymph node metastasis, there were a total of 86 cases (10.24%) in the POLE subgroup, a total of 241 cases (28.69%) in the MSI subgroup, a total of 164 cases (19.52%) in the CNH subgroup, and a total of 348 cases (41.43%) in the CNL subgroup. The results are shown in **Table 2**.

**Figure 8:**
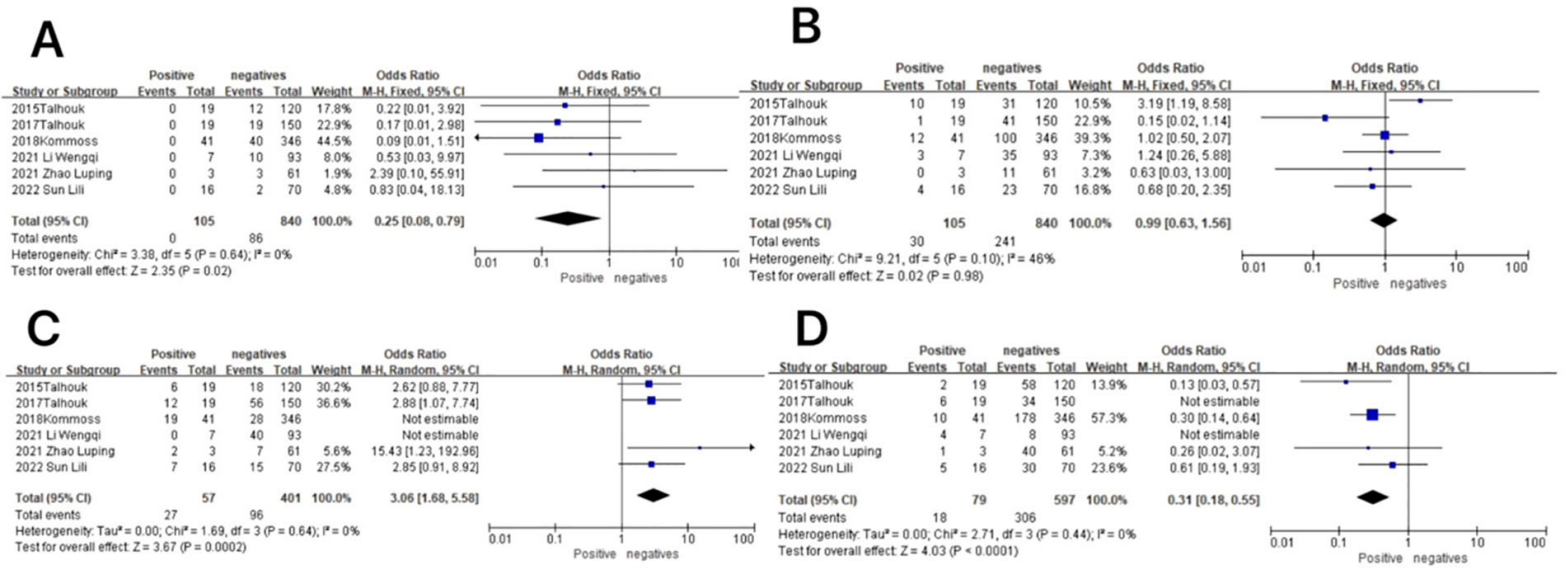
Relationship between molecular typing and lymph node metastatic status in endometrial cancer. **(A)** The POLE subgroup, **(B)** the MSI subgroup, **(C)** the CNH subgroup, and **(D)** the CNL subgroup.

In the POLE subgroup, the heterogeneity test showed that there was homogeneity of results (*I* ^2^ = 0%, *P* = 0.64), so meta-analysis was performed using the fixed-effects model. The results showed that in the POLE subgroup, between patients with lymph node metastasis and without lymph node metastasis (OR = 0.25, 95% CI 0.08–0.79, *P* = 0.02), the diamond shape after observing the combined effect sizes did not intersect with the null line, which indicated that the prevalence of the POLE subgroup was low in patients with lymph node metastasis as compared to those without lymph node metastasis, and the difference was statistically significant. The results are shown in **Figure 8A**.

In the MSI subgroup, the heterogeneity test showed that there was homogeneity of results (*I* ^2^ = 46%, *P* = 0.10), so meta-analysis was performed using the fixed-effects model. The results showed that in the MSI subgroup, between patients with and without lymph node metastasis (OR = 0.99, 95% CI 0.63–1.56, *P* = 0.98), the diamond shape after observing the combined effect size intersected with the null line, which indicated that the results were not statistically significant. The results are shown in **Figure 8B**.

In the CNH subgroup, the heterogeneity test showed that there was moderate heterogeneity in the results (*I* ^2^ = 70%, *P* = 0.005), and the use of the one-by-one exclusion method revealed that the heterogeneity was significantly reduced by removing the articles of Wenqi Li and Kommos (*I* ^2^ = 0, *P* = 0.64) and that the prevalence of the CNH subgroup was higher in patients with lymph node metastasis compared to those without lymph node metastasis (OR = 3.06, 95% CI 1.68–5.58, *P* = 0.0002), and the diamond shape after observing the combined effect sizes did not intersect the null line, indicating that the prevalence of the CNH subgroup was high in patients with lymph node metastasis compared to those without lymph node metastasis, and the difference was statistically significant. The results are shown in **Figure 8C**.

In the CNL subgroup, the heterogeneity test showed that there was high heterogeneity of results (*I* ^2^ = 80%, *P* = 0.001), which was found to be significantly reduced by removing the articles of Wenqi Li and 2015 Talhouk using the one-by-one exclusion method (*I* ^2^ = 0, *P* = 0.44). The results showed that among the CNL subgroup, between patients with and without lymph node metastasis (OR = 0.31, 95% CI 0.18–0.55, *P* < 0.0001), the diamond shape after observing the combined effect sizes did not intersect with the null line, indicating that there was a low prevalence of the CNL subgroup among the patients with lymph node metastasis as compared to those without lymph node metastasis and that the difference was statistically significant. The results are shown in **Figure 8D**.

### 3.5 Publication bias and sensitivity analysis

**Figure 9:**
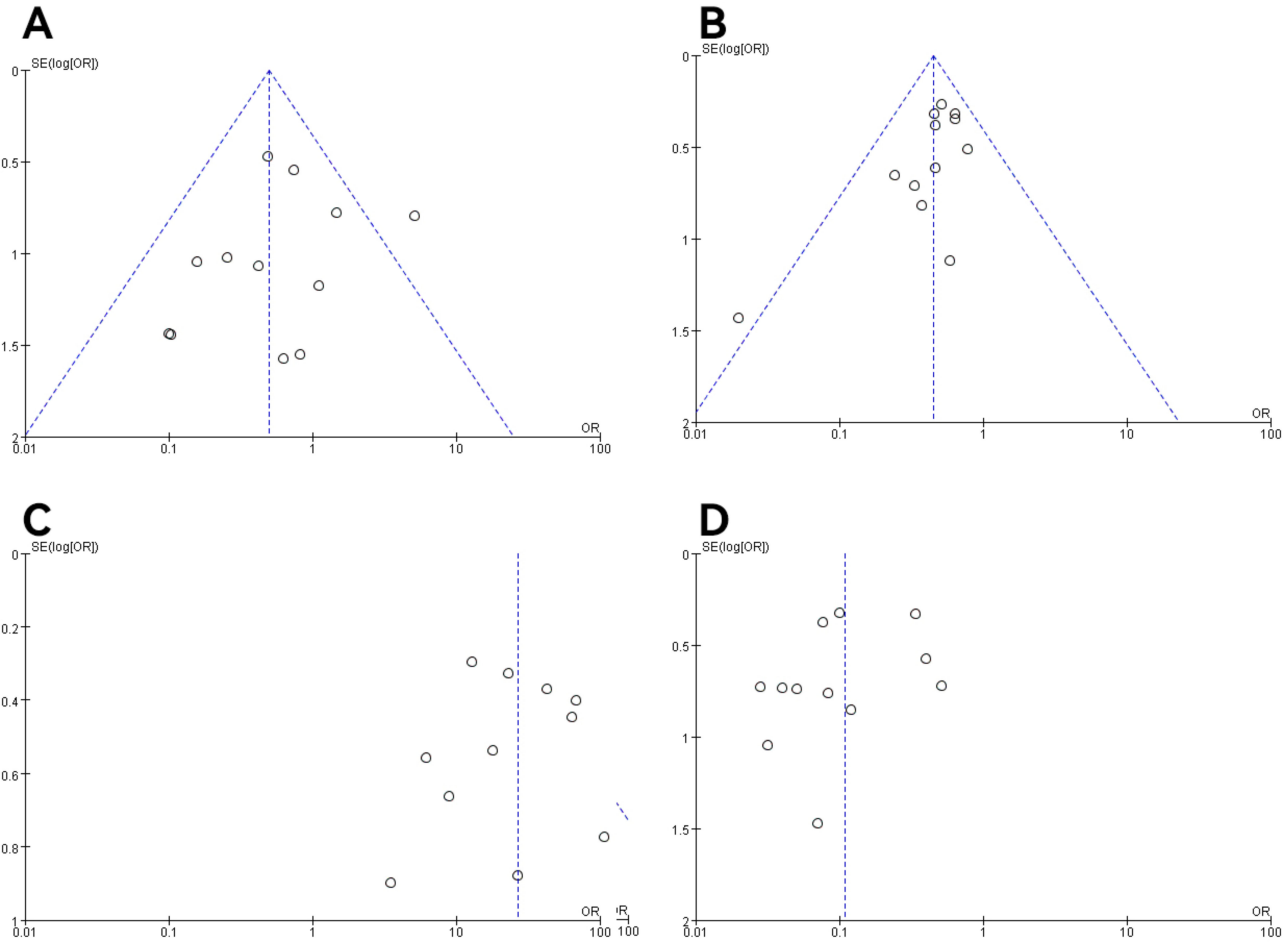
Funnel plot of molecular typing and histological type study of endometrial cancer. **(A)** The POLE subgroup, **(B)** the MSI subgroup, **(C)** the CNH subgroup, **(D)** the CNL subgroup.

**Figure 10:**
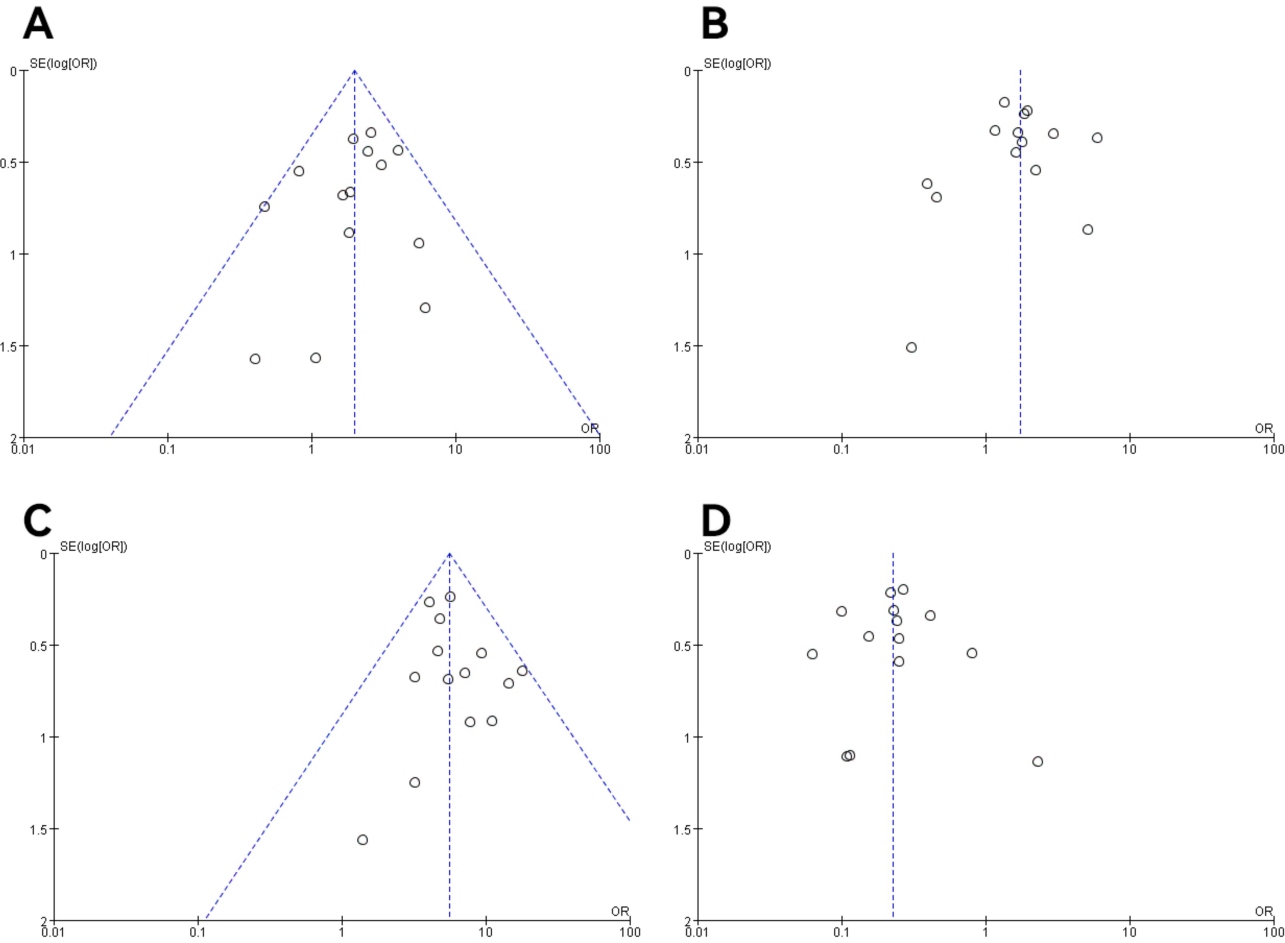
Funnel plot of molecular staging and pathological grading studies of endometrioid carcinoma. **(A)** The POLE subgroup, **(B)** the MSI subgroup, **(C)** the CNH subgroup, and **(D)** the CNL subgroup.

In all studies, publication bias was assessed by plotting funnel plots for studies with >10 included papers. The graphs on both sides of the dotted line of all the funnel plots were basically symmetrical. The publication bias was small and bias had less impact on this analysis. The results are shown in **Figures 9**, **10**.

Sensitivity analyses were performed on studies with >2 articles included in the literature, and the results of the sensitivity test showed that we compared the values of the combined effect sizes of the fixed-effects model and the random-effects model by converting the values of the fixed-effects model and the random-effects model, and the combined ORs and 95% CIs fluctuated within the range of homogeneity of the single studies removed from each study, and the combined effect sizes of the risk factors were close to one another, and it was found that the fixed-effects model was within the confidence interval of the random-effects model, and there was no change in the difference. No differential change occurred, and the results of the meta-analysis were stable, with low sensitivity and good stability. The results of the meta-analysis were stable, with low sensitivity and good stability.

## 4 Discussion

### 4.1 Pathologic characteristics of patients with endometrial cancer in the POLE subgroup

Among the four TCGA subgroups of endometrial cancer, the POLE subgroup is the least common and is also seen to be the least represented in our statistics. Researchers found that this subgroup was mainly present in EEC. The Peking Union Medical College Hospital (18) analyzed 43 patients with the POLE subgroup and found that 86% of patients in the POLE subgroup were in FIGO stage I and that the type of postoperative adjuvant therapy did not affect the survival of patients with POLE hypermutated ECs in FIGO stages I–II; therefore, these data may support the omission of additional adjuvant therapy for such patients. Similarly, in our study, the prevalence of the POLE subtype was significantly higher in FIGO stage I (7.20%) than in FIGO stages II–IV (2.77%), a finding that is consistent with that of Hoa et al. (19). Due to the small number of included studies, Hoa et al. did not find differences in the prevalence of the POLE subgroup in other pathologic features, but they found that patients with the POLE subtype were more likely to be present in the depth of myometrial invasion <50% (6.06%) and without lymph node metastasis (10.24%). In summary, the POLE subgroup was more likely to occur in early-stage endometrial cancer and in some patients with favorable prognostic pathologic features. However, the prevalence of this subgroup was lower in G1–2 EC (5.62%) but significantly higher in G3 EC (10.87%) (OR = 1.98), a finding that is consistent with Travaglino et al. (20) (OR = 2.13, *P* = 0.0001). It has been speculated that the correlation between POLE mutations and high level may be based on the high mutation load in this subgroup (1). However, the association is not fully understood. Therefore, it is necessary to identify patients with G3 EEC who exhibit POLE hypermutation and to further determine the reasons for the greater susceptibility to this mutation in G3 EEC. This finding has important implications for patient management, as the POLE subgroup consistently has the best prognosis of all TCGA subgroups of endometrial cancer (6, 21–24). The 2021 ESGO/ESTRO/ESP guideline (3) categorizes patients with POLE hypermutated EC in FIGO stages I–II without residual lesions as a low-risk group. Notably, researchers found that the POLE subgroup was the only subgroup with a lymph node metastasis rate of 0.00%, a finding that could serve as one of the reasons to support the exceptionally good prognosis of the POLE subgroup, considering the important prognostic value of lymph node involvement (25). Moreover, it implies that according to the 2016 ESMO risk assessment system (26), some G3 EEC patients undergoing lymph node dissection who belong to the POLE subgroup were overtreated, and even unnecessary adjuvant treatments can be avoided. Therefore, it is necessary to perform POLE mutation detection in some G3 EEC patients to avoid overtreatment. In our study, there was no statistically significant difference between the POLE subtype and LVSI in EC patients.

### 4.2 Pathologic characteristics of endometrial cancer patients in the MSI subgroup

The MSI subgroup is the second most common TCGA subgroup in endometrial cancer. Our findings showed that the prevalence of the MSI subgroup was intermediate between the CNL and CNH groups in all unfavorable histopathologic factors. Patients in the MSI subgroup were predominantly present in EEC. The prevalence of the MSI subgroup increased significantly from G1–2 EEC (31.70%) to G3 EEC (42.92%), making it the most common subgroup in G3 EEC. Nearly half of G3 EEC and LVSI were present in the MSI subgroup, thus implying that a high percentage of patients are categorized as high risk according to the 2016 ESMO risk assessment system (26). Considering the moderate prognosis of the MSI subgroup, these patients may be currently overtreated. The prevalence rates of myometrial invasion depth (35.49%, 38.87%) and lymph node metastasis (28.69%, 28.57%) were also similar, but the difference in their respective prevalence was small (*P* > 0.05). Consistent with the CNH subtype, etc., these outcomes are representative of the overall performance of the MSI subtype. The prevalence of myometrial invasion depth and lymph node metastasis of MSI subtype tumors was not significant, and it is not possible to say whether the G1/2 EEC of MSI would be as aggressive as the G3 MSI EEC. Indeed, the MSI subgroup consistently has an intermediate prognosis regardless of histotype, which leads to a worsening of the prognosis of early low-grade EC (27) rather than an improvement in the prognosis of endometrioid EC (6, 28, 29). According to this view, differences in grading and histotype may be part of the morphological heterogeneity of EC in the MSI subgroups but have no impact on prognosis (3). Although massive LVSI (>1 lesion) has been described in all molecular EC groups, an association between massive LVSI and MMRd has been noted, with one study showing that LVSI significantly worsened the prognosis of EC in the MSI subgroup and that the prevalence of massive LVSI was as high as 8.9% in patients with EC in the MSI subtype (*P* = 0.002) (30). On the other hand, LVSI found in our study was predominantly present in the MSI subtype (42.10%). However, researchers did not find statistically significant differences in the prevalence of MSI subgroups at different FIGO stages, depth of myometrial invasion, and lymph node metastasis.

### 4.3 Pathologic characteristics of patients with endometrial cancer in the CNH subgroup

The CNH subgroup has the worst prognosis of the four TCGA subgroups (1). Although the CNH subgroup accounts for only approximately 15% of all endometrial cancer cases, it contributes to 50%–70% of endometrial cancer mortality (31). This subtype differs from the others in that it is significantly present in NEEC (67.69%) and a minority in EEC (6.44%), and overall, the CNH subgroup is the most strongly associated with NEEC (OR = 26.76). Researchers found that the CNH subgroup was very rare in G1–2 EEC (4.70%), whereas the prevalence of the CNH subgroup was significantly increased in G3 EEC (21.17%). TCGA showed that approximately 25% of G3 EEC were combined with serous carcinoma due to TP53 mutations and high copy number alterations. Although the majority of CNH subgroup endometrial cancers are serous, researchers now know that this class of endometrial cancer can be encountered in all histologic types. One study (31) showed that approximately 92.6% of serous carcinomas, 38% of clear cell carcinomas, and 85% of carcinosarcomas were of the CNH subtype. Furthermore, prognostic differences do exist between different histological tumors of this subtype. For example, serous carcinomas may be more aggressive than p53 abnormal endometrioid carcinomas but less aggressive than carcinosarcomas. However, these differences have not been consistently reported, and it is unclear whether they require different treatments. A retrospective study (6) has shown that CNH subtypes are more frequently diagnosed in advanced disease. In our study, researchers also found that the CNH subgroup had a significantly higher prevalence of FIGO II–IV (25.39%), myometrial invasion depth ≥50% (18.83%), LSVI (22.38%), and lymph node metastasis (43.81%). However, the query as to whether the molecular features of EC patients with the CNH subtype appear only at a late stage of disease progression and whether they are associated with the progression from G1–2 to G3 has not yet been answered. In the present study, the pathological grading of EEC in CNH subtypes was obtained mainly to reach a late G3 state, with late adverse prognosis such as increased myometrial invasion, lymph node involvement, and other factors which are represented by the overall presentation of CNH subtypes, suggesting that both the G3 and G1/2 grading can express an adverse prognosis. By the prevalence profile, MSI had the highest percentage of late G3 and G1/2 was CNL-graded. Increased myometrial invasion over 50% had the highest percentage of CNL followed by MSI, and the highest percentage of CNH was only in the case of lymph node metastasis positivity, which could not be illustrated in comparison to the G1/2-graded EEC MSI, CNL, or POLE mutated tumors for G1/2 EEC pathologically graded CNH tumors are also high staging tumors.

In conclusion, the finding that the CNH subtype is more likely to be present in patients with pathologic features of poor prognosis is consistent with the poor prognosis of the CNH subgroup, implying that the CNH subgroup can be considered high-grade cancer regardless of clinicopathologic factors.

### 4.4 Pathologic characteristics of patients with endometrial cancer in the CNL subgroup

The CNL subgroup is the most common subgroup of endometrial cancer (1). It is also known as the “endometrioid subgroup” because it consists mainly of prototypically well-differentiated EC. As expected, researchers found that 50.20% of the EEC and only 10.19% of the NEEC belonged to the CNL subgroup, whereas the CNL subgroup constituted the majority (57.98%) of the G1–2 EEC, which was the only subgroup significantly associated with low FIGO grade. Although the prevalence of the CNL subgroup in G3 EEC was significantly lower, it was still not low (25.04%), second only to the MSI subgroup. Researchers also found that the CNL subgroup was the most common subgroup in both FIGO stage I (47.89%) and FIGO stages II–IV (36.82%), as well as in myometrial invasion <50% (46.76%) and myometrial invasion 50% (39.19%), and that its prevalence was significantly increased in both FIGO stage I and myometrial invasion <50%. Although the CNL subgroup of patients is abundant in low-risk pathologic features, it does not mean that it has the best prognosis among the four subgroups. Some scholars analyzed the survival prognosis of the four subgroups in G3 EEC and found that the prognosis of the CNL subgroup was worse than that of the MSI subgroup and even similar to that of the CNH subgroup in NEEC (32). Combined with previous studies in which differences in different pathologic features in the TCGA subgroup were examined in this paper, it can be shown that some patients may be over- or undertreated if only one of the risk assessment criteria (clinicopathologic features or molecular features) is used. In our report, for the CNL subgroup, its prevalence was second only to the MSI subgroup in the LVSI and the medium-high EMSO (2016) clinicopathologic stratification system. In conjunction with previous studies, the CNL subgroup does not yet have a precise prognostic value closely related to its wide distribution in both of the above-described pathologic features. Since the prognosis of the CNL group ranges from good to intermediate, a molecular-based classification may have little impact on the management of patients in this group. The CNL subgroup lacks molecular/immunohistochemical features and therefore requires the absence of molecular features of the other three subgroups to be defined (1). Thus, in the absence of further molecular and prognostic stratification of the CNL subgroup, clinicopathologic factors remain critical for assessing risk in this group. Therefore, molecular and prognostic substratification of this subgroup is a priority for future research.

### 4.5 Strengths and limitations

To the best of our knowledge, this study is the first meta-analysis in China and abroad to comprehensively assess the variability in the prevalence of the four molecular subtypes of endometrial cancer across different pathologic features. Because of the limited range of populations currently studied for the molecular staging of EC, this paper used meta-analysis to pool data from a large number of populations to observe the variability of the prevalence rates of the four TCGA subgroups in histologic type, FIGO grade, FIGO stage, LVSI, depth of myometrial invasion, and lymph node status. The fact that researchers included articles that had to include all four subtypes at the same time had overlooked some of the data from articles that performed pathological characterization of a single subtype, but at the same time, researchers used sensitivity analysis to ensure the robustness of each result. Our article did not perform a further meta-analysis of molecular staging prognosis because the surgical modalities and adjuvant therapies used in the included populations varied widely in clinical prognostic studies of molecular staging and were difficult to standardize, so it is a limitation of the article that researchers provide guidance for clinical studies by comparing the differences in the distribution of the four subtypes across pathologic features and combining them with prognostic conclusions provided by other studies.

## 5 Conclusion

1. Patients in the POLE subgroup were more likely to be found in early-stage endometrial cancer and had pathologic features with better prognosis (EEC, myometrial invasion depth <50%, no lymph node metastasis), and the lymph node metastasis rate of patients in the POLE subgroup was extremely low. However, patients in the POLE subgroup were more likely to be found in G3 EEC than in G1–2 EEC, suggesting that some G3 EECs are susceptible to overtreatment based on clinical diagnosis only based on pathology and that POLE mutation testing should be performed in such patients, which can narrow the scope of surgery (e.g., lymph node dissection) and reduce unnecessary adjuvant therapy after surgery.
2. Patients in the MSI subgroup were more likely to be found in EEC and G3 EEC as well as LVSI; nearly half of G3 EECs as well as LVSI were present in patients in the MSI subgroup.
3. The CNH subgroup was more likely to be diagnosed to have advanced endometrial cancer and had poor prognostic pathologic features (NEEC, G3 EEC myometrial invasion depth ≥50%, LVSI, and lymph node metastasis), a finding consistent with the poor prognosis of the CNH subgroup.
4. Patients in the CNL subgroup were more likely to be found in early endometrial cancer and had all pathologic features with a favorable prognosis (EEC, G1–2 EEC, myometrial invasion depth <50%, no lymph node metastasis, no LVSI). Furthermore, the CNL subgroup was present in large numbers in both early and advanced endometrial cancers, and the lack of a precise prognostic value of the CNL subgroup was closely linked to its wide distribution in all pathologic features. The CNL subgroup does not yet have a precise prognostic value and its wide distribution across all pathologic features is closely related. Clinicopathologic factors remain critical for assessing risk in this group, and molecular and prognostic substratification of this subgroup is a focus for future research.

## Data Availability

The original contributions presented in the study are included in the article/supplementary material. Further inquiries can be directed to the corresponding author.
